# Giant Ovarian Cyst in a Term Pregnancy Simulating a Massive Ascites: A Case Report

**DOI:** 10.7759/cureus.33199

**Published:** 2022-12-31

**Authors:** Adeola Olasinde, Olumuyiwa Ogunlaja, Yetunde T Olasinde, Mojisola U Mobolaji-Ojibara, Nafisat Adelaja-ojulari

**Affiliations:** 1 Obstetrics and Gynaecology, General Hospital, Ilorin, NGA; 2 Obstetrics and Gynaecology, Bowen University, Iwo, NGA; 3 Pediatrics and Child Health, Bowen University, Iwo, NGA

**Keywords:** case report, pregnancy, cystadenoma, ascites, giant ovarian cyst

## Abstract

The diagnosis of ovarian mass during pregnancy may be missed if early ultrasonographic assessments of pregnancy are not done, resulting in late detection and management. Hence, this case report is aimed at underscoring the possibility of misdiagnosis of a giant ovarian mass for massive ascites in pregnancy. We present the case of a 35-year-old unbooked multigravida woman who was referred to our centre on account of polyhydramnios in labour, misdiagnosed as a massive ascites on admission. The incidental intraoperative diagnosis of a right giant ovarian cyst resulted in the right salpingo-oophorectomy of a huge cyst with the histological diagnosis of a benign ovarian serous cystadenoma. Huge cystic ovarian tumours coexisting with a normal pregnancy may simulate massive ascites resulting in a misleading diagnosis as reported in this case study. We report this case to awaken the suspicion index of a giant ovarian cyst in any patient presenting with massive ascites in pregnancy.

## Introduction

The occurrence of coexisting ovarian cysts in pregnancy is not uncommon in pregnancies where there are acceptable diagnostic imaging techniques [1]. The broad application of obstetrics ultrasound in antenatal care services has significantly enhanced the early detection of adnexal cysts in pregnancy and consequently provides important clues for clinical decision-making [2].

Ovarian cysts are diagnosed in 0.05-5% of pregnancies [3], which are mostly small size and benign [4]. However, less than 1% of all ovarian cysts in pregnancy are giant ovarian cysts [5] and may sometimes be unrecognized or misdiagnosed [5,6].

Ovarian cysts coexisting with pregnancy are usually asymptomatic and incidentally detected during ultrasound assessment of early trimester pregnancy, which may resolve spontaneously [7]. However, giant ovarian cysts may present with progressive abdominal distention and pressure symptoms such as urinary retention, respiratory embarrassment, easy satiety, vomiting, and constipation [8].

 Few reported cases of giant ovarian cysts were misdiagnosed as ascites [8-10]. This case is reported to increase the suspicion index of giant ovarian cysts in pregnancy presenting as massive ascites.

## Case presentation

We present the case of a 35-year-old female (gravida 5, para 4+4 alive) at an estimated gestational age of 38 weeks and six days. She was a trader with a primary level of education, referred from Civil Service Hospital, in Ilorin, Nigeria (a comprehensive health centre).

Upon referral, she was admitted to the labour room of General Hospital, Ilorin, Nigeria with complaints of intermittent lower abdominal pain and liquor drainage per vaginum three hours before presentation. The indication for referral was ultrasound confirmation of polyhydramnios in labour with attending potential foetal risk and need for neonatal care. She was unbooked and presented at the referring centre with an ultrasound result showing severe polyhydramnios at term (amniotic fluid index of 46 centimetres) with sudden onset of liquor drainage resulting in her transfer. Her past obstetrics and medical histories were normal.

On general examination, her vitals were stable. The abdomen was grossly distended with difficulty in delineating the foetal parts and palpating for uterine contraction. There was relative palpable fundal height at 34 centimetres with a positive fluid thrill in the upper quadrants of the abdomen above the fundus of the uterus and flanks fullness raising suspicion of severe ascites. The foetal heart sound heard with foetal Sonicaid (Huntleigh Healthcare Ltd, Cardiff, Wales) was 148 beats per minute. From pelvic examination, she was in the active phase of first-stage labour on admission with cervical dilatation of 6cm, 80% effacement, vertex presentation, station 0, and absent foetal membranes.

Laboratory tests including complete blood count, electrolytes, urea and creatinine, random blood sugar, and viral marks (hepatitis and HIV) were within normal limits. A repeat obstetric ultrasound scan on admission revealed a viable foetus with massive ascites.

She had augmentation of labour with oxytocin infusion but had poor progress of labour despite adequate contraction and, hence, underwent emergency caesarean section under region anaesthesia. An alive female baby of 2.7 kg was delivered with good APGAR scores at the first and fifth minutes. There were no noticeable ascites but a giant right-sided ovarian cyst extending to the epigastric region after exteriorizing the uterus. The uterine incision was closed in two layers with absorbable Vicryl 2. An attempt at exteriorizing the cyst through the Pfannenstiel incision was impossible.

The cyst tension was decompressed after suctioning about three litres of cyst contents (clear, straw colour, odourless fluid) using a venous cannula needle perforation (cystostomy) for ease of exteriorizing the cyst through the Pfannenstiel incision site. The cyst wall was elevated with two clamps to prevent leakage of the cyst fluid. The needle was then removed and an incision of 0.5cm in diameter was made at the same place. An aspirator tip was placed through that incision to reduce the cyst size and the 0.5cm wide cut that was made to the cyst wall was ligated. When the authors were aspirating the cyst fluid, controlled reperfusion was performed by the anaesthesiology team to prevent persistent hypotension during the operation. The decompressed right giant ovarian cyst exteriorized, a right salpingo-oophorectomy was done, and the sample was sent for routine histological evaluation.

**Figure 1:**
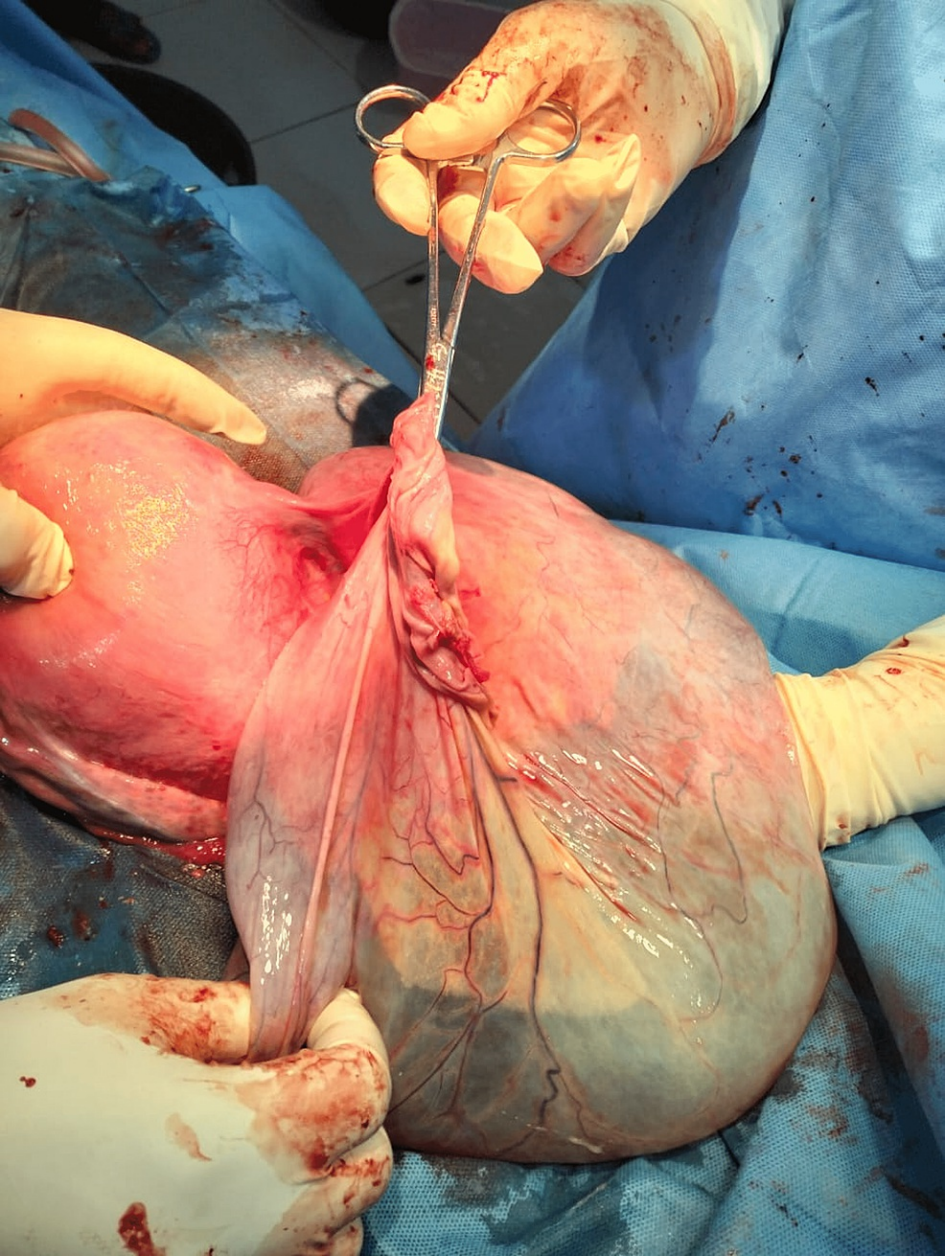
Right giant ovarian cyst being exteriorized and resected during Caesarean section at term with live baby weighing 2.7 kg

The exploration of the lower abdomen showed that the mass was originating from the right ovary. The left ovary and other pelvic organs were normal in appearance. The total weight of the aspirated cyst content and removed cystic mass was 5.7 kg. The abdomen was closed in layers. Figure 1 shows the exteriorized right giant ovarian cyst that was resected. 

The postoperative period was uneventful, and the patient was discharged from the hospital on the third day after surgery. No complications were observed in the six weeks after surgery. The histopathological study of the specimen reported a serous cystadenoma of the ovary.

## Discussion

The occurrence of giant ovarian cysts in pregnancy simulating ascites has been seldomly reported in the literature [1,11]. Despite the use of ultrasonography as the mainstay of diagnosis of coexisting ovarian cysts in pregnancy with a high level of accuracy [1,10], this is not without misdiagnosis. In our case report, the provisional diagnosis was massive ascites in pregnancy and polyhydramnios at term, which were contrary to the intraoperative finding of a giant ovarian cyst in pregnancy. 

Ovarian cyst coexisting with pregnancy has various manifestations, often non-specific pressure symptoms, such as back or abdominal pain, constipation, abdominal distension, and urinary complaints [7]. Since these issues are generally attributed to regular problems of pregnancy, they may be neglected by either the patient or the physician. The patient in this report had no unusual symptoms different from what she considered normal in pregnancy and did not book pregnancy in the hospital until labour commenced. This attributed to late detection and intervention. 

Some patients in our setups seek antenatal care late due to illiteracy, favourable previous pregnancy experiences, and financial constraints but this is not without risks of morbidity and mortality when complications develop. The non-specific clinical features in ovarian cysts mimicking some features of pregnancy lead to the challenges of late diagnosis and delayed intervention if no diagnostic imaging is done [10].

Many giant ovarian cysts can present with signs and symptoms of ascites due to their large nature and they are commonly mistaken for it, with the flanks fullness, and demonstrable shifting dullness on abdominal examination [9]. This was typical in this reported case, and the initial sonographic evaluations failed to diagnose the giant ovarian cyst correctly.

Intraoperative diagnosis of giant ovarian cysts may be inevitable where there are preoperative misdiagnoses, and the inappropriate choice of abdominal incision may create surgical challenges as in this patient.

## Conclusions

The presence of very large cystic masses in pregnancy may masquerade as ascites resulting in a misleading diagnosis and altering the mode of treatment. The ultrasonographic examination of giant ovarian cysts in pregnancy is not without misdiagnosis if not thoroughly evaluated. Hence, giant ovarian cysts should always be considered in the differential diagnosis of large ascites in pregnancy for appropriate intervention. The role of antenatal care in this instance cannot be overemphasized.
